# Polymeric Carriers for Delivery of RNA Cancer Therapeutics

**DOI:** 10.3390/ncrna8040058

**Published:** 2022-08-02

**Authors:** Sofía Mirón-Barroso, Joana S. Correia, Adam E. Frampton, Mark P. Lythgoe, James Clark, Laura Tookman, Silvia Ottaviani, Leandro Castellano, Alexandra E. Porter, Theoni K. Georgiou, Jonathan Krell

**Affiliations:** 1 Department of Surgery and Cancer, Imperial College, London W12 0HS, UK; a.frampton@imperial.ac.uk (A.E.F.); m.lythgoe@imperial.ac.uk (M.P.L.); j.clark@imperial.ac.uk (J.C.); l.tookman@imperial.ac.uk (L.T.); j.krell@imperial.ac.uk (J.K.); 2 Department of Materials, Imperial College London, London SW7 2AZ, UK; j.correia@imperial.ac.uk (J.S.C.); a.porter@imperial.ac.uk (A.E.P.); t.georgiou@imperial.ac.uk (T.K.G.); 3 Department of Clinical and Experimental Medicine, University of Surrey, Guildford GU2 7XH, UK; 4 Department of Biosciences, Nottingham Trent University, Nottingham NG1 4FQ, UK; silvia.ottaviani@ntu.ac.uk; 5 School of Life Sciences, University of Sussex, Brighton BN1 9RH, UK; l.castellano@sussex.ac.uk

**Keywords:** RNA cancer therapeutics, polymeric carriers

## Abstract

As research uncovers the underpinnings of cancer biology, new targeted therapies have been developed. Many of these therapies are small molecules, such as kinase inhibitors, that target specific proteins; however, only 1% of the genome encodes for proteins and only a subset of these proteins has ‘druggable’ active binding sites. In recent decades, RNA therapeutics have gained popularity due to their ability to affect targets that small molecules cannot. Additionally, they can be manufactured more rapidly and cost-effectively than small molecules or recombinant proteins. RNA therapeutics can be synthesised chemically and altered quickly, which can enable a more personalised approach to cancer treatment. Even though a wide range of RNA therapeutics are being developed for various indications in the oncology setting, none has reached the clinic to date. One of the main reasons for this is attributed to the lack of safe and effective delivery systems for this type of therapeutic. This review focuses on current strategies to overcome these challenges and enable the clinical utility of these novel therapeutic agents in the cancer clinic.

## 1. Introduction

Cancer is a leading cause of death worldwide and a major healthcare challenge [1]. Traditional cancer treatments, such as chemo or radiotherapy, target rapidly proliferating cells in a non-specific manner. Healthy cells, not only cancer cells, are affected and this can result significant undesirable off-target effects for patients. In addition, primary and secondary resistance can lead to poor response or tumour relapse [2].

As research uncovers the underpinnings of cancer biology [3], new targeted therapies have been developed. The majority of these targeted therapies are small molecules, such as kinase inhibitors [4], which work by targeting active sites in proteins involved in tumour development and cancer progression. However, only 1% of the genome encodes for proteins and only a subset of these proteins has ‘druggable’ active binding sites [5]. Another class of targeted therapy are recombinant proteins, such as monoclonal antibodies that target cancer-specific epitopes or aberrant post-translational modifications in cancer cells [6]. Recombinant proteins present certain restraints such as their instability and complex and expensive manufacturing requirements that involve folding and post-translational modifications [7].

## 2. RNA Therapeutics for Cancer Treatment

In the last decades, RNA therapeutics have gained popularity due to their ability to affect targets that small molecules cannot. Additionally, they can be manufactured more rapidly and cost-effectively than small molecules or recombinant proteins. RNA therapeutics can be synthesised chemically and altered quickly, which can enable a more personalised approach to cancer treatment [8].

There are several modalities of RNA therapeutics with potential in the cancer clinic. Synthetic mRNA technology can be employed to develop cancer vaccines that elicit an immune response against specific tumour epitopes [9,10]. Antisense oligonucleotides can be designed to inhibit the translation of specific mRNAs that encode for proteins involved in tumour development and progression [11].

Some RNA therapeutics take advantage of the endogenous mechanisms of RNA interference including small interfering RNAs (siRNAs) and microRNAs (miRNAs). siRNAs can be artificially introduced to bind with base complementarity and inhibit the translation of a specific mRNA involved in tumour development and progression [12]. On the other hand, miRNAs are endogenous molecules that can regulate the expression of multiple mRNAs involved in tumorigenesis [13,14]. Synthetic miRNA therapeutics that can either mimic or inhibit miRNAs are being developed as potential treatments in the cancer clinic [15].

Aptamers are single-stranded oligonucleotides that have a specific three-dimensional structure that allows them to bind to specific target molecules with high affinities. Aptamers have the potential to replace monoclonal antibodies because they present less immunogenicity and have an easier and a more cost-effective manufacturing process [16,17].

Even though a wide range of RNA therapeutics are being developed for various indications in the oncology setting, none has reached the clinic to date. One of the main reasons for this is attributed to the lack of safe and effective delivery systems for this type of therapeutic.

### 2.1. Need for Delivery Systems

As RNA molecules are hydrophilic and negatively charged, they do not easily cross biological membranes which have a hydrophobic section and a negatively charged surface. Furthermore, endo- and exo-nucleases present in biological fluids can rapidly degrade RNA. Foreign RNA can trigger the innate immune response via the activation of Toll-like receptors, which have evolved to recognise microbial infections by sensing extrinsic nucleic acid [18]. Even though activation of the immune response might be beneficial in some cases, such as vaccines or immuno-therapeutics, it can be detrimental for other indications. Moreover, the undesirable pharmacokinetic profile of RNA therapeutics can hinder their ability to reach their required site of action because of their short half-life due to rapid degradation and renal clearance.

Some progress has been made to overcome these barriers. These include chemical modifications in synthetic RNA, such as using phosphorothioates as analogues of the phosphate backbone, incorporating methylated nucleobases, introducing alterations of the ribose 2′ hydroxyl group [19,20,21]. These modifications can confer resistance to degradation by nucleases, increasing the half-life of the RNA therapeutics as well as decreasing their immunogenicity. However, RNA therapeutics are still unable to cross biological membranes and are rapidly cleared by the kidneys. Thus, there is still a need to develop and optimise systems for RNA delivery.

### 2.2. Gene Delivery Systems

Viral vectors are the most widely studied systems for the delivery of gene therapeutics. Recent developments have been made in this field, particularly the use adeno-associated viruses (AAV) to improve tropism for certain target tissues [22]. However, their limited packaging capacity [23] and safety issues, especially related to their immunogenicity, have hindered their translation into the clinical setting. Furthermore, viral vectors are expensive and difficult to manufacture and scale up.

Lipid-based delivery systems have also been widely studied for the delivery of RNA therapeutics. In fact, several products have reached the market including Patisiran, the first iRNA therapeutic approved by the FDA [24] and the recently developed vaccines against SARS-CoV-2 [25,26]. However, lipid-based delivery systems have difficulty reaching target tissues because they of their low specificity and tendency to accumulate in the liver. They can be administered locally, such as in the case of vaccines, or used to target liver conditions, such as Patisiran; however, further progress needs to be made to deliver RNA therapeutics to other target organs.

Several types of inorganic nanoparticles have also been studied for the delivery of RNA therapeutics for cancer treatment. For instance, mesoporous silica nanoparticles with tuneable pore sizes and surface chemistry have been developed. These nanoparticles have large surface areas in the pores that can be modified by adding positive charges which enable the encapsulation of nucleic acids. Furthermore, nanoparticle surfaces can be also modified to incorporate targeting moieties and specific ligands [27,28,29]. Another type of inorganic nanoparticles used to delivery RNA are gold nanoparticles. Gold nanoparticles present several advantages such as unique optical properties, high biocompatibility and precise synthesis with controlled size and shape [30,31]. However, inorganic nanoparticles are not biodegradable, and their accumulation can lead to long term toxicity. Thus, more studies are necessary to prove their safety profile in in vivo models.

Extracellular vesicles are secreted by mostly all cell types containing biomolecules such as DNA, RNA, proteins or lipids to deliver information to other cells. Their natural biocompatibility makes them ideal candidates as delivery systems for external RNA therapeutics. However, their production process is complex and difficult to scale up [32,33].

Other methods to deliver RNA therapeutics to cancer cell are physical methods, which include sonoporation, particle bombardment and laser-assisted nucleic acid delivery. These methods present low immunogenicity However, they can cause tissue damage, lack selectivity and require knowledge of the precise location of the tumour.

## 3. Polymeric Carriers

Polymeric carriers have been widely studied for the delivery of RNA therapeutics because of their versatility, potential multi-functionality and relative low cost. Polymers are macromolecules that can be defined by different characteristics such as their composition, architecture, molecular mass or charge [34].

### 3.1. Polymer Composition

**Figure 1:**
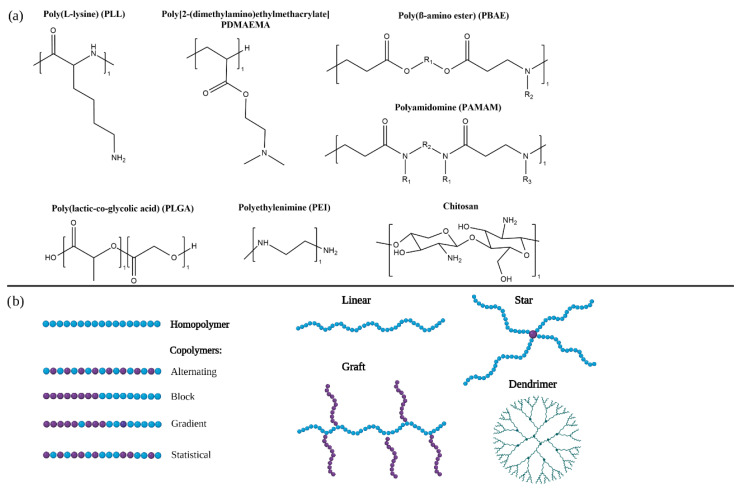
(**a**) Chemical structures of commonly used polymers in RNA therapeutics. (**b**) Schematical illustrations of different polymer architectures and topologies.

A variety of polymers are being developed for the delivery of RNA therapeutics (Figure 1a, Table 1. They can be classified in homopolymers, composed of only one type of monomer, or co-polymers if they include several types of monomers (Figure 1b).

The most widely studied cationic polymer for RNA delivery is **polyethyleneimine (PEI)** due to its high transfection efficiency. Its primary, secondary and tertiary amines are protonated at physiological pH and enable nucleic acid complexation, cellular internalisation and endosomal escape. However, PEI presents high toxicity and immunogenicity that has hindered its translation into the clinic. Combination of PEI with poly(ethylene glycol) [35] or hydrophobic moieties such as cholesterol [36] is being studied to decrease its toxicity and enable a safe and effective delivery of RNA therapeutics.

**Chitosan** is a naturally sourced polysaccharide widely studied for RNA delivery due to its biocompatibility, biodegradability, low toxicity and immunogenicity. Furthermore, the ability to fine-tune several of its parameters such as the degrees of deacetylation (DDA) or its charge by altering the fractions of protonatable amine has made it appealing for the development of gene delivery systems [37]. This cationic co-polymer is composed of β-linked N-acetyl glucosamine and D-glucosamine, and its amino groups are protonated at physiological pH which allows it to interact with negatively charged nucleic acids [38]. However, these interactions with nucleic acids are not very strong and can cause premature release and low efficiency; several strategies are being developed to overcome these issues [39].

**Poly(L-Lysine) (PLL)** is a biodegradable homopolymer which contains primary amines that can be protonated to interact with RNA but can cause toxicity in vivo. Novel architectures, such as PLL dendrigrafts, are being developed to deliver RNA therapeutics [40]. Approaches to reduce PLL toxicity such as complexation with anionic compounds are being studied [41].

**Poly(lactic-*co*-glycolic acid) (PLGA)** is a copolymer composed of lactic and glycolic acid, widely used for drug delivery. It is FDA approved, biodegradable and biocompatible. Its tuneable properties, such as the ratio of lactic acid to glycolic acid, enable the controlled release of encapsulated therapeutics. Systems based on PLGA are being developed for the delivery of RNA therapeutics [42,43]. The combination of PLGA with cationic polymers such as PEI are being studied to improve RNA condensation [44].

**Polyamidoamine (PAMAM)** dendrimers have also been developed for delivery of RNA [45]. Strategies, such as grafting targeting moieties, are being studied to increase their selectivity towards diseased cells [46,47]. Higher dendrimer generations lead to higher efficacy, but also increased toxicity; the balance between these parameters is key in the design of PAMAM gene delivery systems [48].

**Poly(β-amino esters) (PBAE)** are biodegradable and biocompatible polymers that can be easily modified. The application of PBAE for RNA delivery is being studied. However, there is a need to optimise the balance between their toxicity and efficiency in vivo [49] as well as their stability in order to accomplish their translation into the clinic [50].

**Poly[2-(dimethylamino)ethyl methacrylate] (PDMAEMA)** is a promising polymer for delivery of RNA therapeutics. It contains tertiary amines that interact with RNA and allow endosomal escape and cellular internalisation [51,52,53].

A common co-monomer that is often introduced to cationic polymer chains is **poly(ethylene glycol)** (PEG) because of its biocompatibility. It is present in the formulation of many FDA approved products, such as the COVID-19 vaccines. Thus, many studies reported that by introducing PEG or PEG based monomers like oligo(ethylene glycol) methyl ether methacrylate (OEGMA) resulted in decreased toxicity and prolonged circulation time [51,52,53,54].

### 3.2. Polymer Architectures

In copolymers, monomers can be arranged in different manners which can results in statistical, alternating, gradient and block copolymers. The effect of the different arrangement of monomers on gene delivery efficiency is being studied [55]. Statistical copolymers that include cationic and non-ionic or anionic monomers have reported higher efficacy and toxicity than block copolymers with the same composition. This might be due to the lack of a hydrophilic block that hinders interaction with cellular membranes. However, block copolymers were observed to have increased colloidal stability probably due to the steric hindrance of the hydrophilic blocks [56,57,58].

Polymers can also present different spatial architectures (Figure 1B). In linear polymers monomers are only bond to one or two other monomers. Incorporation of crosslinkers that bind more than two monomers can result in different architectures such as stars, grafts, branched polymers or dendrimers [55].

Branched architectures have been shown to increase efficiency over linear polymers [59]. They include branched copolymers in which secondary polymer chains are linked to a primary backbone and dendrimers [34].

Dendrimers consist of a central core and highly branched arms. They are synthesised in a controlled manner and are characterised by their generation which refers to the number of branches additions. With each generation the volume and surface increase as well as the number of terminal groups. Generally, dendrimers are characterised by a very narrow size distribution. The most commonly used dendrimers for gene delivery are poly(amidoamine) (PAMAM) [45,46,47,48] and poly(propylenimine) (PPI) [60,61] dendrimers.

Another architecture emerging for promising delivery systems for nucleic acids are star copolymers. They consist of several linear homo- or co-polymers bond to a core forming a star shaped structure [62,63,64,65]. Star shaped polymers have reported higher transfection efficiencies than their linear counterparts which can be due to a higher condensation of the nucleic acids [66].

### 3.3. Molecular Mass

Molecular mass distribution of polymers is one of the most studied characteristics. Increasing molecular mass have generally shown to increase efficiency and cytotoxicity [67]. This can be due to the increase of the probability of interaction with cellular membranes. Molecular mass distribution can also impact the ability of polymers to escape the endosome. Higher molecular mass polymers reported increased endosomal escape [68]. Optimizing the molecular mass to balance efficiency and toxicity is a key consideration in the design of polymeric delivery systems [59].

### 3.4. Polyplexes Formulation

The formation of polyplexes is mostly driven by electrostatic interactions. A key parameter in polyplex formulation is the N/P ratio (the ratio of nitrogen groups of the polymer to the phosphate groups of the nucleic acid). Higher N/P ratios lead to higher transfection efficiency and colloidal stability due to the electrostatic repulsion of the positive charges in the surface of the polyplexes. However, high N/P ratios can also cause toxicity as a result of the interactions of the polymer’s positive charges with negatively charged proteins and cellular membranes [69].

Other preparation methods, such as the buffer used or the mixing of reagents, can have an influence on the physicochemical characteristics of the polyplexes and ultimately their transfection efficiency. Mixing the reagents by pipetting instead of dropwise addition leads to lower hydrodynamic diameters and narrower size distributions, as well as lower transfection efficiency [70].

### 3.5. Characterisation Techniques

In order to reach the clinical setting, polyplexes need to be thoroughly characterised. **Size** is one of the key parameters that has a great impact on the pharmacokinetic profile of polyplexes. Several techniques have been developed to evaluate the size distribution of nanosised systems.

Dynamic light scattering (DLS) determines the hydrodynamic diameter of the polyplexes by relating it to their Brownian motion using the Stokes–Einstein equation. DLS is ideal to determine the hydrodynamic diameter distribution of mono-population, nanosised particles. Fluorescent correlation spectroscopy (FCS) is also used measure the size and diffusion coefficient of fluorescently labelled polyplexes [71].

Atomic force microscopy (AFM) allows the visualisation particles’ surface and morphology at high resolutions scanning the sample with a cantilever tip. Scanning electron microscopy (SEM) is used to determine the surface, morphology and composition by creating images from the scattered electrons. Transmission electron microscopy (TEM) provides information on the inner structure, size and morphology as well as on the cellular internalisation of the polyplexes. It creates images from the electrons transmitted through the sample [72].

The **charge** at the surface of the polyplexes can be determined by their zeta potential. The zeta potential can be measured by electrophoretic mobility, observing how the particles move when an electric field is applied. This parameter is crucial for the polyplexes’ stability as well as its’ safety and efficiency [73].

The **molecular mass** and **composition** are also key parameters for polymer characterisation. Gel permeation chromatography (GPC) is the standard method for determining the molecular mass. Nuclear magnetic resonance (NMR) spectroscopy can also be used to determine the polymer’s molecular mass as well as to accurately determine monomer composition for copolymers [74]. Fourier transform infrared spectrometry (FTIR) can also be used to characterise polymers and determine their composition [75].

## 4. Barriers for Polymeric Carriers

### 4.1. Protein Corona, Opsonisation and the MPS

Several barriers must be overcome to allow successful delivery of polymeric carriers to their site of action. Some relate to their route of administration. For systemic administration, one of the biggest concerns is the absorption of proteins to the surface of nanoparticles [76]. Polymeric carriers are generally positively charged and, thus, proteins, which are commonly negatively charged, can bind through electrostatic interactions.

The absorption of proteins causes the formation of a protein corona surrounding the nanoparticles. This protein corona can change the physicochemical characteristics of the nanoparticles such as their size, charge and surface chemistry. These properties greatly affect their pharmacokinetic profile and biological activity [77]. Furthermore, some of these proteins can be opsonins, including immunoglobulins, coagulation and complement proteins [78]. Opsonins are recognised by the mononuclear phagocyte system (MPS) which mainly includes Kupffer cells present in the liver and spleen macrophages. Opsonins can mark nanoparticles and trigger their phagocytosis and elimination, as well as cause changes in their biodistribution and promote accumulation in organs such as the liver or spleen. Opsonisation can prevent nanoparticles from reaching their site of action, as well as trigger an immune response causing severe side effects [79].

Extracellular anionic glycosaminoglycans (GAG) can also displace nucleic acids and lead to a prompt release of the therapeutic agent before reaching it site of action [80].

Furthermore, the formation of this protein corona in the surface of nanoparticles can hide targeting moieties, such as aptamers or antibodies, and, thus, hinder their ability to target specific organs or cell types [81].

Nevertheless, binding of certain proteins, such as albumin, can allow nanoparticles to evade the immune system and can increase targeting to tumour cells. Albumin accumulates in the tumour due to the leaky vasculature present in the tumour tissue and is known that cancer cells take up plasma proteins in a higher rate than normal cells and utilise their degradation products for proliferation [82,83].

A widely studied strategy to overcome this barrier is PEGylation. Grafting poly(ethylene glycol), a hydrophilic polymer, to the surface of nanoparticles to block the absorption of proteins by steric hindrance and shields the positive charges from the surface, thereby improving the biodistribution to target organs [84]. However, several recent studies have reported the production of antibodies against PEG upon repeated administrations of PEGylated nanoparticles and that pre-existing anti-PEG antibodies can lead to accelerated clearance of PEGylated nanoparticles and reduced efficiency [85]. Several approached to overcome this issue are being developed, such as using free PEG molecules to saturate anti-PEG antibodies [86] or grafting nanoparticles with alternative hydrophilic molecules [87].

### 4.2. Tissue Targeting

**Figure 2:**
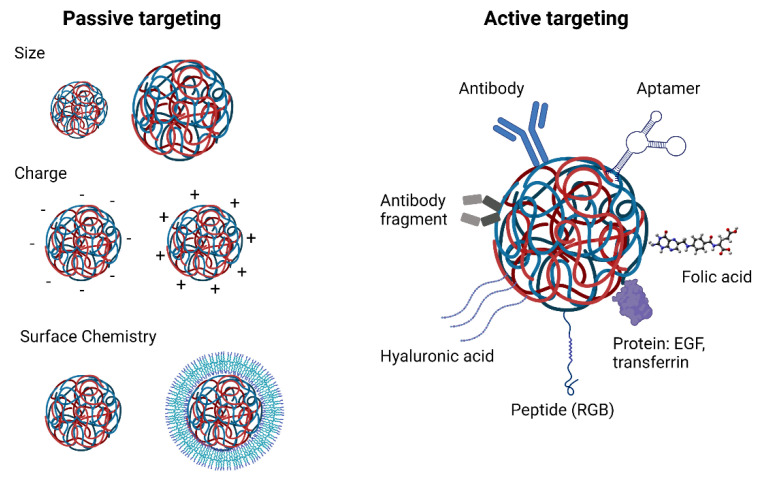
Active and passive strategies for tissue targeting of polymeric carriers.

Reaching the target tissue is one of the main barriers for the delivery of RNA therapeutics to cancer cells. Targeting strategies are categorised in active or passive (Figure 2). Passive strategies rely on characteristics of the delivery system. Different physicochemical properties of polymeric nanoparticles such as their size, charge and surface chemistry greatly affect their biodistribution [88]. Nanoparticles smaller than 6 nm can be quickly excreted by the kidneys. [89]. On the other hand, nanoparticles with a hydrodynamic diameter larger than 150 nm are prone to be taken up by phagocytic cells in the spleen. Furthermore, nanoparticles tend to accumulate in the liver due to the fenestrated vasculature of the liver sinusoids and can be eliminated by the MPS [90]. Rapid renal clearance and liver accumulation decrease the nanoparticle’s half-life reducing the possibility of the nanoparticles to reach their site of action. Thus, choosing an appropriate nanoparticle size that is not too small to be quickly excreted by the kidneys and not too large to be quickly taken up by the MPS is key in designing an optimal delivery system.

Moreover, a widely studied, but controversial, strategy for passive targeting of nanoparticles to solid tumours is the Enhanced Permeation and Retention (EPR) effect. The EPR effect was firstly described by Maeda in 1986 [91]; he observed that macromolecules tended to accumulate in tumours due to their abundant vasculature, defective blood vessels with increased permeability and the lack of efficient lymphatic drainage. Since his discovery, many studies have been performed using this strategy to target drug delivery systems to solid tumours. However, results have revealed large variability of this effect in vivo and in human patients [92]. In murine models, tumour blood vessels do not develop properly due to the rapid growth of tumour xenografts and, thus, have higher number of fenestrations and are leaky to nanoparticles. However, tumours in humans grow slower than in murine models and the vasculature is not as permeable, which decreases the efficiency of the EPR effect. Furthermore, this variability might be due to the heterogeneity of tumour tissue, and factors such as the tumour tissue of origin, tumour size and vascularisation can modulate the EPR effect. Many solid tumours present a high intratumoural interstitial fluid pressure due the high vascularisation and impaired lymphatic drainage as well as a dense extracellular matrix composed of which a network of collagen, proteoglycans, elastin fibres and hyaluronic acid, which can hinder the transport of nanoparticles into tumours [93,94]. However, this phenomenon is still an important strategy used for targeting polymeric delivery systems to primary tumour and metastasis [95,96].

Different strategies based physicochemical characteristics of nanoparticles are being developed to improve targeting of non-viral vectors to specific tissues. In a recent study, SORT (Selective Organ Targeting) was developed to engineer lipid nanoparticles to selectively target certain organs [97].

Active targeting, which involves the grafting of specific moieties to the surface of nanoparticles, is the most well-studied strategy to accomplish selective tissue targeting of polymeric nanoparticles to date. These ligands include peptides such as RGD (arginine, glycine, aspartic acid) which binds selectively to α_v_β_3_ integrins generally overexpressed in tumour vasculature endothelial cell [98,99,100], as well as antibodies, antibody fragments or aptamers that recognise certain surface receptors that are overexpressed in cancer cells such as HER2 [101,102,103]. Other molecules used for active targeting of polymeric nanoparticles to tumours are transferrin [104,105], folic acid [106,107], hyaluronic acid [108,109] and epidermal growth factor (EGF) [110] due to the overexpression of their receptors in cancer cells [111].

Active targeting allows nanoparticles to be internalised more efficiently by a specific cell type. However, the interaction between ligands and receptors only occurs when both molecules are within a very short distance of each other. Active targeting does not lead to tumour accumulation, but it improves selective cell uptake. Hence, a combination of both strategies is ideal when designing delivery systems. Passive targeting can enable nanoparticles to reach tumours and active targeting can trigger nanoparticles internalisation in cancer cells.

In order to reach cancer cells within tumours nanoparticles must cross the endothelium. In brain tumours, such as glioblastoma or brain metastasis, this barrier becomes harder to cross. The blood–brain barrier (BBB) formed by endothelial cells attached to each other by tight junctions hinders the transport of drugs to the brain. Several strategies are being developed to enable nanoparticles to cross the BBB and deliver drugs to the brain such as grafting transferrin to the nanoparticles surface to target the transferrin receptor [112] or using penetrating peptides that target lipoprotein receptors [113], both of which are overexpressed in the BBB.

### 4.3. Cellular Uptake

Once nanoparticles reach the tumour, they need to be internalised by cancer cells. Most polymeric nanoparticles are made of cationic polymers that interact with negatively charged nucleic acids If the net charge of the polyplexes is positive, these nanoparticles can be internalised by binding via electrostatic interactions to the negatively charged glycocalyx in the cell membrane in a non-specific manner [114].

Moreover, targeting moieties on the surface of nanoparticles can trigger cellular uptake by receptor-mediated endocytosis. There are different endocytosis pathways that can be involve in nanoparticle internalisation: clathrin-mediated, caveolae-dependent, macropinocytosis and clathrin- and caveolae- independent pathways [115].

When nanoparticles are internalised by most of these pathways they will be transported to the endo-lysosomal compartment. Internalised nanoparticles are entrapped in vesicles which gradually become early endosomes, late endosomes and, finally, lysosomes. During this process protons are pumped into the vesicles causing the pH to decrease. The acidic pH and the presence of hydrolases in the lysosomal compartment can degrade RNA therapeutics and thus dramatically decrease treatment efficacy.

### 4.4. Endosomal Escape

**Figure 3:**
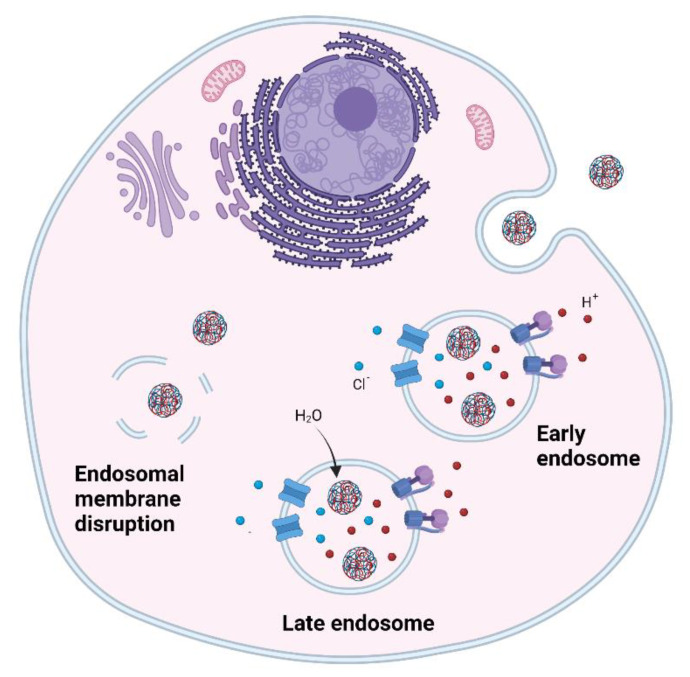
Endosomal escape. Proton sponge effect.

Endosomal entrapment is a huge bottleneck in the delivery of RNA therapeutics and their translation to the clinic. It has been observed that certain polymers such as PEI are able to escape the endosome, however the precise mechanism is not entirely known. One well-known hypothesis is the proton sponge effect (Figure 3) [116,117]. This hypothesis states that polymers containing high number of amino groups have high buffering capacity and act as proton sponges. The high influx of protons into the endosomes causes a flow of chloride atoms that cause an indirect entry of water in the endosome. The high osmotic pressure disrupts the endosomal membrane and causes the release of the polyplexes. However, after many years of research this hypothesis has not been verified and alternative hypothesis have been proposed, such as the direct membrane permeabilisation hypothesis. This hypothesis states that there is a charge-driven interaction of polyplexes with the endo-lysosomal membrane which causes the formation of transient holes and increases its permeability remaining the endosome intact [118].

Several polymer properties, such as their molecular mass or/and pKa, can impact their ability to escape the endosome. Higher molecular mass polymers reported increased endosomal escape [68] and polymers with a pKa ranging from 5.8 to 6.2 showed increase efficiency in siRNA delivery [119].

### 4.5. Balance between Transfection Efficiency, Toxicity and Immune Activation

Generally, polymers used for RNA delivery are positively charged due to the ability of cationic polymers to interact with negatively charged nucleic acids to form polyplexes as well as with negatively charged cellular and endosomal membranes to allow internalisation and endosomal escape. However, this positive charge can cause cellular membranes disruption of non-targeted cells and interact with negatively charged proteins in biological fluids which can lead to toxicity and immune system activation. Different strategies are being developed to circumvent this issue such as the use of negatively charged coatings [120].

Usually, increasing the positive charge of the polymeric carriers leads to an increased transfection efficacy, but also in toxicity and immune activation. Breaking this correlation is a long standing goal in the field of polymeric gene delivery [121]. However, both transfection efficiency and toxicity are dependent on the cell type [122].

Furthermore, it is not appropriate to directly compare the transfection efficiency of even the same polymer carriers in the same cell lines from different studies because often different transfection protocols and formulations are used.

Size can also play a role on the safety profile of nanoparticles. As mentioned previously, nanoparticles larger than 5 nm are required to avoid renal clearance and increase nanoparticle’s half-life so that they can reach the target tissue. However, accumulation of nanoparticles in certain tissues can cause toxicity. Ideally, nanoparticles should be cleared after delivering the RNA to the targeted tissue. Biodegradable polymers, such as PLGA, PBAE and polycaprolactone (PCL), are being studied to overcome this issue [38,123,124].

### 4.6. Tumour Heterogenicity

An important challenge in the development of polymer gene delivery systems is tumour heterogenicity. Different transfection efficiencies are reported on the same systems when transfecting different cell types [125]. Many different cell types can be found in tumour microenvironments, such as tumour-associated macrophages, cancer-associated fibroblasts, immune cells and endothelial cells [126].

Furthermore, genomic instability in cancer cells causes intratumoural heterogeneity and leads to the presence of different cancer cell clones with different properties, which can result in different transfection efficacy of the same polymeric carrier [3,127].

## 5. Smart Polymeric Carriers

**Figure 4:**
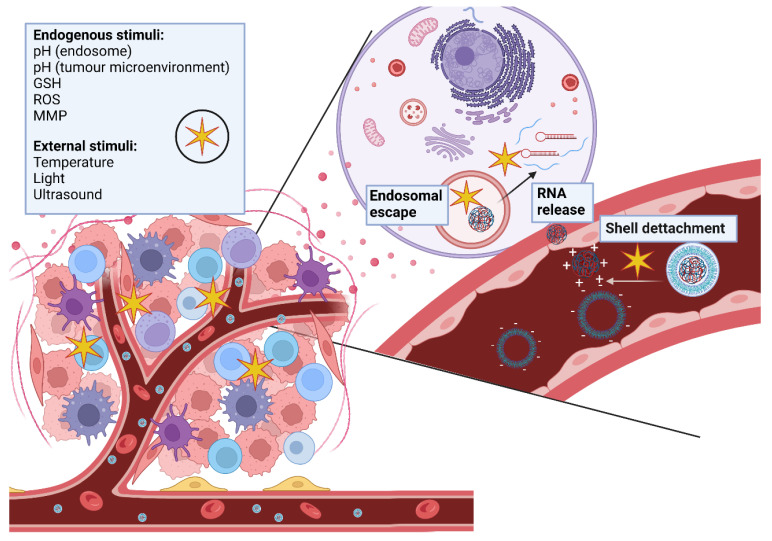
Smart polymeric nanocarriers respond to endogenous and exogenous stimuli which trigger shell detachment, endosomal escape and RNA release into the cytoplasm.

Polymeric nanoparticles have great potential to deliver RNA therapeutics for cancer treatment. However, as previously described there are still limitations that must be overcome. In order to do so, researchers are developing smart polymeric nanocarriers that are able to sense and react to internal or external stimuli (Figure 4).

One of the main endogenous stimuli being exploited is the acidic pH of the endo-lysosomal compartment. To avoid degradation of RNA therapeutics in the lysosome and enable endosomal escape, pH-responsive polymers that disassemble and are able to disrupt membranes at endosomal pH (5–6) are being developed [128,129]. pH-responsive polymers have also been designed to undergo disassembly and membrane disruption in response to the slightly acidic pH of the tumour microenvironment. These polymers become protonated at pH 6.8, in contrast to the physiological pH 7.4, and expose targeting moieties or cell-penetrating peptides to allow internalisation into cancer cells [130,131].

Tumour tissue is also characterised by a high level of reactive oxygen species (ROS). Polymeric nanoparticles with ROS-cleavable linkages that break and allow the release RNA in the presence of ROS are being developed to increase selectivity to tumour tissues [132,133,134].

Another endogenous stimulus that allows to control over the release of the encapsulated drug is the redox state. The difference between the high intracellular concentrations of glutathione (GSH) (2–10 mM) compared to that of the extracellular environment (2–20 µM) can be used to trigger drug release only when the nanoparticle has reached the cytoplasm. Polymeric nanoparticles containing disulphide links that can be reduced by intracellular glutathione are being developed to avoid prompt release of therapeutics in the extracellular space [135,136].

Ideally, nanoparticles should have a negatively charged surface to prolong circulation time and allow them to reach their target tissue but having a positive charge enables cellular uptake. In a recent study, the development of polymeric nanoparticles with a negatively charged shell linked by a pH-sensitive bond was described. This bond breaks when the nanoparticles reach the slightly acidic tumour microenvironment exposing a positively charged core triggering cellular internalisation. The core of these polymeric nanoparticles is linked by redox-sensitive bonds and is able to dissociate in the cell cytoplasm releasing the drug [137].

Approaches using the activity of specific enzymes that are overexpressed in the tumour microenvironment such as matrix metalloproteinases (MMP) to increase selectivity are being studied [138]. Polymeric nanoparticles with PEG grafted on their surface via an MMP-sensitive peptides have been developed. These nanoparticles lose their PEG coating in an MMP rich environment, such as the tumour tissue, exposing their cationic core that encapsulates siRNA or targeting moieties which enable cellular internalisation [139,140].

External stimuli can also be used to trigger RNA delivery to tumours. One of the most common stimuli is temperature; mild hyperthermia can be induced in tumours via different techniques such as infrared light. A moderate increase of temperature has been reported to promote blood flow and increase vascular permeability as well as make cancer cells more sensitive to therapeutics. Mild hyperthermia can be used as a trigger for temperature-responsive polymers to release the encapsulated drug to tumour tissues [141,142]. Other external stimuli used to facilitate tumour targeting and controlled drug release are ultrasound [134,143,144] and light [145,146].

## 6. Conclusions

RNA therapeutics can enable targeted and personalised approaches and, thus, hold great promise as cancer therapeutics. However, due to the instability and suboptimal pharmacokinetics of RNA molecules, there is a significant need for safe and effective delivery systems before they can reach the clinic.

The versatility and multi-functionality of polymeric carriers make them ideal candidates to enable the delivery of RNA therapeutics. Even though there are many biological barriers that polymeric carriers need to overcome to reach the site of action, significant advances are being made in this field. These include an improved understanding of the interaction between polymers and the biological environment including serum proteins and the immune system, as well as their interaction with cancer cells. Furthermore, advances in polymerisation and characterisation techniques have resulted in greater control over the engineering and design of polymeric carriers. Finally, the design and development of smart polymeric carriers able to sense and react to different stimuli are allowing for increased RNA delivery efficiency while maintaining optimal safety profiles.

## Figures and Tables

**Table 1:** Polymers for RNA delivery.

Polymer	Advantages	Limitations	Ref.
PEI	High transfection efficiency	High toxicity and immunogenicity	[35,36]
Chitosan	Biocompatibility, biodegradability, low toxicity and immunogenicity	Premature release and low transfection efficiency	[37,38,39]
PLL	Biodegradability, high transfection efficiency	Toxicity	[40,41]
PLGA	FDA approved, biodegradability and biocompatibility	Low efficiency	[42,43,44]
PAMAM	Dendrimers highly efficiency	Toxicity	[45,46]
PBAE	Biodegradability and biocompatibility	Limited ability to sustain delivery over long timespans, toxicity	[49,50]
PDMAEMA	High transfection efficiency	Non-biodegradable	[52,53]

## Abbreviations

RNA ribonucleic acid, mRNA messenger RNA, miRNA microRNA, siRNA small interfering RNA, AAV adeno-associated viruses, PEI polyethyleneimine, DDA deacetylation, PLL poly(L-Lysine), PLGA Poly(lactic-co-glycolic acid), PAMAM Poly(amidoamine), PBAE Poly(β-amino esters), PDMAEMA Poly[(2-(dimethylamino)ethyl methacrylate)], OEGMA oligo(ethylene glycol)methyl ether methacrylate, PEG polyethylene glycol, MPS mononuclear phagocyte system, GAG glycosaminoglycans, BBB blood–brain barrier, PCL Polycaprolactone, ROS reactive oxygen species, GSH glutathione, MMP matrix metalloproteinases.
